# Influences of professional self-concept and job stress of nurses working in Korea dementia care centers on turnover intention

**DOI:** 10.3389/fpubh.2025.1573586

**Published:** 2025-06-18

**Authors:** Mi Young Kim, Minkyung Gu, Nam Kyung Oh, Sohyune Sok

**Affiliations:** ^1^Department of Nursing, Graduate School, Kyung Hee University, Seoul, Republic of Korea; ^2^Department of Nursing, College of Health Science, Daejin University, Pocheon-si, Gyeonggi-do, Republic of Korea; ^3^College of Nursing Science, Kyung Hee University, Seoul, Republic of Korea

**Keywords:** turnover intention, job stress, professional self-concept, dementia, nurse

## Abstract

**Background:**

As the older adult population increases, the prevalence of dementia is increasing. The number of nurses working in dementia care centers is increasing, and related research is needed for them.

**Objective:**

This study aimed to examine the influences among professional self-concept, job stress, and turnover intention, and the factors influencing turnover intention of nurses working at dementia care centers.

**Methods:**

A cross-sectional explanatory survey with path analysis was employed. Participants were 160 nurses working in dementia care centers in South Korea. Measures were the general characteristics list, the Professional Self-Concept of Nurses Instrument, the job stress scale, and turnover intention measurement instrument.

**Results:**

Turnover intention was positively correlated with job stress (*r* = 0.35, *p* < 0.01), and it was negatively correlated with professional self-concept (*r* = −0.42, *p* < 0.01). The job stress (*β* = 0.53, *p* < 0.001), age (*β* = −0.22, *p* = 0.048), educational level (*β* = 0.19, *p* = 0.014), and professional self-concept (*β* = −0.19, *p* = 0.023) were statistically significant factors influencing turnover intention (explanatory power: 21.0%).

**Conclusion:**

Strategies or interventions relieving job stress and strengthening professional self-concept could decrease turnover intention. Age and educational level need to be considered when developing and implementing interventions. These results were similar to the results of studies on the turnover intention of hospital nurses. However, the results of this study are meaningful in the reality that there is a great lack of research on the turnover intention of nurses working at dementia care centers in community.

## Introduction

In Korea, the prevalence of dementia is rapidly increasing due to the increase in the older adult population, which is expanding into a regional disease burden. According to a study on the older adult population, the number of dementia patients aged 65 years or older is continuously increasing from 6.8% in the 1990s, 8.8% in 2010, 9.7% in 2020, and 10.4% in 2022 (1). Such an increase in the prevalence of dementia has raised the value of supporting individuals and families with dementia patients, highlighting the promotion of the “management project for dementia care center” as a pressing issue of the Korean national government. In this regard, the work functions of nurses in dementia care centers are also gradually expanding (2).

In dementia care centers, nurses and nursing assistants make up the majority of the main management project staff. Specifically, nurses make up the largest percentage at approximately 55.6% (3). Dementia care center nurses perform a relatively high level of work in the scope of primary and tertiary prevention of dementia (4, 5). In particular, unlike clinical nurses, they perform various roles such as early screening, treatment and screening cost support, education and support operation for dementia patients and their families (2, 5). As the scope of work for dementia care center nurses diversifies, enhancing their capacity is essential for improving their professional self-concept in caring for dementia patients (4).

For dementia care center nurses, professional self-concept can enhance job satisfaction and nursing performance, ultimately maximizing their capacity to care for dementia patients professionally (6). Nurses in dementia care centers with positive and firm professional self-concept can resolve internal work problems (7). In addition, they can perform their role more efficiently and effectively, benefitting dementia patients and themselves as they maintain their job embeddedness (8, 9). Studies show that this professional self-concept can reduce their turnover rate (7–9). On the other hand, nurses in dementia care centers with negative professional self-concept tend to lack the ability to perform nursing care for dementia patients due to low confidence in their work and a lack of communication skills and flexibility. Job stress may also further increase due to their maladaptation in nursing work and conflicts with employees in other departments (10, 11). Nurses working at dementia care centers often complain of job stress due to the diverse and difficult tasks (8). Consequently, there is a high turnover rate of nurses in dementia care centers (8, 9). Hence, job stress from pursuing work efficiency and developing a professional self-concept are crucial in increasing nurses’ job satisfaction, which contributes to securely maintaining their positions in dementia care centers (7, 12). As the older adult population in Korea rapidly increases and the prevalence of dementia increases, the work functions of nurses in dementia care centers are gradually expanding. Therefore, it is urgent to establish measures for the continuous and stable management of the nursing staff in dementia care centers (13). Although there are studies on professional self-concept and job stress as factors related to turnover intention among nurses working in general hospitals (7, 14–16), there is still a lack of studies on nurses working in dementia care centers in the community (9, 13).

The purpose of this study was to examine the influences among professional self-concept, job stress, and turnover intention, and the factors influencing turnover intention of nurses working at a dementia care center. The concrete aims were to (1) identify the general and job-related characteristics of nurses at the dementia care center; (2) examine the levels of the professional self-concept, job stress, and turnover intention of dementia care center nurses; (3) examine the correlations between professional self-concept, job stress, and turnover intention of dementia care center nurses; (4) examine the impact of professional self-concept and job stress of nurses at a dementia care center on turnover intention.

### Theoretical foundation

**Figure 1:**
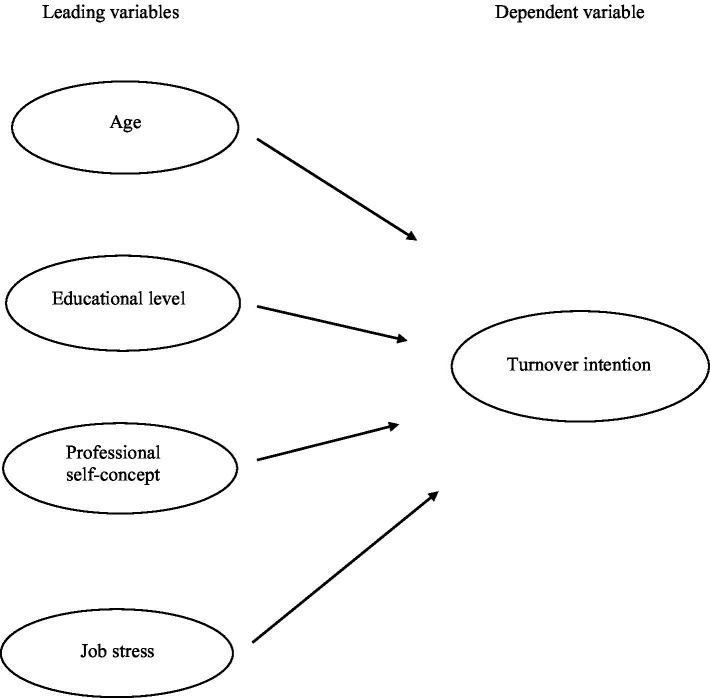
Conceptual framework of this study.

Job Demands-Resources (JD-R) Theory, developed by Bakker and Demerouti (17), is a theory that explains employee well-being and performance through job-related demands and resources. This theory consists of Job Demands and Job Resources. Job Demands refer to the physical, psychological, social, or organizational aspects that employees face while performing their jobs. These demands drain employees’ energy, and high levels of job demands can lead to stress and burnout. Examples include excessive workload, time pressure, and role conflict. Job Resources refer to the physical, psychological, social, or organizational aspects that support and motivate job performance and increase job satisfaction. Job resources can mitigate the negative effects of stress and promote personal growth and development. Examples include support from supervisors, job autonomy, and career development opportunities. JD-R Theory suggests that the balance between job demands and resources has a significant impact on employee well-being. Self-Concept Theory was theoretically developed by Rogers (18), who emphasized the importance of self-concept along with person-centered therapy. This theory explains the thoughts and evaluations that an individual has about himself, that is, the self-concept. Self-concept consists of Self-Image, Self-Esteem, and Ideal Self. Self-concept has a significant influence on an individual’s behavior, motivation, and emotions. Therefore, the theoretical framework of this study was established based on the Job Demands-Resources (JD-R) Theory or Self-Concept Theory. For the conceptual framework of this study, general characteristics (age, educational level), professional self-concept, and job stress were selected as leading variables that are likely to affect the turnover intention of nurses working in dementia care centers (Figure 1). Turnover intention is affected by general characteristics (age, educational level), professional self-concept, and job stress. As hypotheses, H1) Turnover intention is affected by age among general characteristics of nurses. H2) Turnover intention is affected by educational level among general characteristics of nurses. H3) Turnover intention is affected by professional self-concept. H4) Turnover intention is affected by job stress.

## Methods

### Study population

This study employed a cross-sectional explanatory survey design, and path analysis via multiple linear regression was conducted to assess the influence of exogenous variables. The study participants were nurses working in dementia care centers in South Korea. The following are the inclusion criteria considered in the research: nurses who had clear consciousness and understood the study’s purpose and nursing managers in dementia care centers. New nurses with less than 3 months of work experience were excluded from this study because it might be difficult for them to provide direct and effective care to patients, which would affect the reliability of the results.

The sample—136 people—was acquired using the G*Power 3.1.9 program, with a significance level (⍺) of 0.05, a power (1-*β*) of 0.90, and an effect size of 0.15 (19). Considering that a 20% dropout rate would occur, 170 questionnaires were disseminated. Then, 160 questionnaires were collected (94.12% collection rate) and included in the final data. Since the sample size of this study may differ in working conditions depending on the dementia care center in each region of Korea, substantial data were collected from the nurses in dementia care centers located in 17 metropolitan cities nationwide.

### Measurements

#### General characteristics of study participants

Study participant’s general characteristics questionnaire consisted of a total of 5 items including gender, age, marital status, total clinical experience, and educational level.

#### Professional self-concept

Professional self-concept was measured using Arthur (20) Professional Self-Concept of Nurses Instrument, translated by Sohng and Noh (21). This instrument consists of three sub-domains: professional practice (flexibility, leadership, skills), communication, and satisfaction. This is answered using a four-point Likert scale, with scores ranging from a minimum of 27 to a maximum of 108 points, with higher scores indicating a higher nursing professional self-concept. The reliability of the instrument was Cronbach’s *α* = 0.85 in Sohng and Noh (21) study, and the reliability of the instrument in this study was Cronbach’s *α* = 0.85.

#### Job stress

Job stress was measured using the instrument utilized by Cheon et al. (22) to assess the level of stress experienced by nurses at work. It consists of 14 questions, including three sub-domains, where five questions are about role conflict, five are related to role ambiguity, and four refer to inhomogeneity. The tool is measured using a five-point Likert scale with a minimum score of 14 to a maximum of 70, where the higher the score, the higher the stress. In this study, the reliability of the instrument was Cronbach’s *α* = 0.87, similar to Cheon et al. (22) study.

#### Turnover intention

Turnover intention was measured using the turnover intention measurement instrument modified and supplemented by Kim (23) according to the actual situation in Korea. This instrument has 11 questions, including four questions on the intention to change in the sub-areas and seven questions on the turnover factor. However, in this study, only four questions on turnover intention were used to confirm only the intention to leave the job. The instrument has a five-point Likert scale, with scores ranging from 4 (minimum) to 20 (maximum), indicating that higher scores imply higher turnover intentions. The reliability of this instrument was Cronbach’s *α* = 0.84 in Kim (23) study, and Cronbach’s α = 0.88 in this study.

#### Data collection

This study collected data from nurses working at dementia care centers located in 17 metropolitan cities across the country from November to December, 2023. The researchers visited the dementia care centers in person to ask for cooperation, distributed the questionnaire only to nurses who voluntarily gave written consent to participate in this study, and asked them to answer the questionnaire in a self-reporting manner.

#### Ethical considerations

This study was conducted after receiving approval from the Kyung Hee University Institutional Review Board (IRB No. KHSIRB-23-429-RA, Approval date October 22, 2023). Nurses at the dementia care center were made aware in advance of confidentiality issues related to provision of personal information and anonymity. The data for this study was placed in the researcher’s personal locker and locked so that it cannot be accessed by anyone other than the researcher. After the study is completed, it is stored for 3 years after the end of the study for future inspection. After the study is finished, all documents are shredded permanently.

#### Data analysis

The collected data was analyzed according to the research purpose using SPSS/WIN 29.0 program. Descriptive statistics and frequency analysis were used to examine the general characteristics of dementia care center nurses. The levels of professional self-concept, job stress, and turnover intention of dementia care center nurses were examined using the mean and standard deviation. Independent *t*-test, ANOVA, and Scheffe post-hoc test were used to examine the differences of professional self-concept, job stress, and turnover intention according to the general characteristics of dementia care center nurses. The correlations between the professional self-concept, job stress, and turnover intention of dementia care center nurses was analyzed using Pearson’s coefficient correlation. Multiple regression analysis was used to examine the impact of the professional self-concept and job stress on the turnover intention of dementia care center nurses.

## Results

### General characteristics of the study participants

The nurses in dementia care centers included 6 males (3.8%) and 154 females (96.3%). In terms of age, 67 (41.9%) were over 40, and 33 (20.6%) were 30–34 years old. One hundred eight (67.5%) were married, and 55 (34.4%) had the highest number of total clinical experience with 5–6 years, followed by 51 (31.9%) with three to 4 years. Lastly, the majority, 95 (59.4%) nurses, had bachelor degree (Table 1).

**Table 1:** General characteristics of the study participants.

Characteristics	n	%
Gender		
Male	6	3.8
Female	154	96.3
Age (year)		
25 >	4	2.5
25–29	25	15.6
30–34	33	20.6
35–39	31	19.4
40 ≤	67	41.9
Marital status		
No	52	32.5
Yes	108	67.5
Total clinical experience (year)		
1–2	33	20.6
3–4	51	31.9
5–6	55	34.4
7 ≤	21	13.1
Educational level		
College	57	35.6
University	95	59.4
Graduate school	8	5.0

### Levels of professional self-concept, job stress, and turnover intention

The most scores on professional self-concept, job stress, and turnover intention of the nurses in dementia care centers were close to the median (Table 2).

**Table 2:** Levels of professional self-concept, job stress, and turnover intention.

Variables	Range	Median	Min	Max	Mean ± SD
Professional self-concept	27–108	74.00	58.00	96.00	73.53 **±** 5.50
Job stress	14–70	42.00	25.00	57.00	41.86 **±** 5.15
Turnover intention	4–20	14.00	4.00	20.00	13.33 **±** 3.23

### Differences on professional self-concept, job stress, and turnover intention according to the general characteristics of study participants

In this study, the professional self-concept showed a statistically significant difference in terms of the total clinical experience (*F* = 2.60, *p* = 0.047). The job stress presented a statistically significant differences in terms of age (*F* = 3.23, *p* = 0.014) and educational level (*F* = 4.56, *p* = 0.012). The turnover intention exhibited a statistically significant difference in terms of educational level (*F* = 2.44, *p* = 0.048) (Table 3).

**Table 3:** Differences on professional self-concept, job stress, and turnover intention according to general and job characteristics of study participants.

Characteristics	Professional self-concept		Job stress		Turnover intention	
	Mean ± SD	Independent *t*-test or F test (*P*) *Scheffe*	Mean ± SD	Independent *t*-test or F test (*P*) *Scheffe*	Mean ± SD	Independent *t*-test or F test (*P*) *Scheffe*
Gender						
Male	70.17 (5.49)	−1.53 (0.127)	41.33 (6.41)	−0.25 (0.801)	13.83 (2.40)	0.39 (0.695)
Female	73.66 (5.48)		41.88 (5.12)		13.31 (3.26)	
Age (year)						
25 >^a^	72.00 (3.16)	2.02 (0.094)	33.75 (3.40)	3.23 (0.014*) a > b,c,d,e	10.75 (3.60)	1.57 (0.185)
25–29^b^	71.64 (8.15)		40.84 (6.18)		13.72 (2.69)	
30–34^c^	73.33 (4.45)		42.12 (4.14)		14.18 (2.76)	
35–39^d^	72.65 (5.17)		41.90 (4.60)		12.84 (2.96)	
40≤^e^	74.84 (4.79)		42.57 (5.18)		13.13 (3.64)	
Married						
No	72.46 (6.52)	−1.72 (0.088)	42.23 (5.59)	0.64 (0.525)	13.60 (3.02)	0.74 (0.462)
Yes	74.05 (4.89)		41.68 (4.95)		13.19 (3.32)	
Total clinical experience (year)						
1–2^a^	71.45 (4.66)	2.60 (0.047*) c,d > a,b	41.18 (4.81)	0.86 (0.465)	13.09 (3.57)	0.32 (0.814)
3–4^b^	73.35 (6.77)		42.14 (5.53)		13.69 (2.80)	
5–6^c^	74.38 (4.61)		42.45 (5.34)		13.18 (3.39)	
7≤^d^	75.00 (4.76)		40.67 (4.14)		13.19 (3.33)	
Educational level						
College^a^	73.63 (5.96)	0.72 (0.487)	43.14 (5.59)	4.56 (0.012*) c > a	13.26 (3.27)	2.44 (0.048*) c > a
University^b^	73.66 (5.10)		41.41 (4.53)		13.16 (3.21)	
Graduate school^c^	71.25 (6.92)		38.00 (6.72)		15.75 (2.43)	

*p < 0.05. a,b,c,d,e = Scheffe post hoc test.

### Correlations among professional self-concept, job stress, and turnover intention

The turnover intention showed a negative correlation with professional self-concept (*r* = −0.42) and a positive correlation with job stress (*r* = 0.35). In other words, the turnover intention increases as professional self-concept decreases and job stress increases (Table 4).

**Table 4:** Correlations among professional self-concept, job stress, and turnover intention.

Variables	Professional self-concept	Job stress	Turnover intention
	r (*P*)		
Professional self-concept	1		
Job stress	−0.37 (< 0.01*)	1	
Turnover intention	−0.42 (< 0.01*)	0.35 (< 0.01*)	1

*P < 0.05.

### Factors influencing turnover intention

Based on the conceptual framework of this study, the result of analyzing the effect on the turnover intention of the nurses in dementia care centers by the regression analysis method revealed that the influencing factors on the turnover intention of the nurses in dementia care centers were: job stress (*β* = 0.53, *p* < 0.001), age (*β* = −0.22, *p* < 0.048), educational background (*β* = 0.19, *p* < 0.014), and professional self-concept (*β* = −0.19, *p* < 0.023) with the final explanatory power of 21.0% in the regression analysis model (Table 5). The study identified that the job stress of the nurses in dementia care centers was high when they were younger, while the nurses’ high educational level and low professional self-concept had a greater effect on their turnover intention. To enhance clarity of this results, we provide a table summarizing the hypotheses tested, including the direction of the relationship, statistical values, and whether the hypothesis was supported (Table 6). Hypotheses H1, H2, H3, and H4 were all supported.

**Table 5:** Factors influencing turnover intention.

Variables	B	S. E.	β	*t*	*P*	95% CI	
						Lower	Upper
Gender	−0.32	1.24	−0.02	−0.26	0.798	−2.77	2.14
Age	−0.59	0.31	−0.22	−1.91	0.048*	−1.20	0.02
Married	1.24	0.77	0.18	1.62	0.108	−0.28	2.76
Total clinical experience	0.30	0.27	0.09	1.11	0.064	−0.23	0.83
Educational level	1.12	0.45	0.19	2.48	0.014*	0.23	2.01
Professional self-concept	−0.11	0.05	−0.19	−2.29	0.023*	−0.20	−0.02
Job stress	0.33	0.05	0.53	6.28	< 0.001*	0.23	0.44

Durbin-Watson = 1.71, Tolerance = 0.39–0.96, VIF = 1.04–2.56. Adj. R^2^ = 0.21, *F* = 5.87, *P < 0.001. B, Unstandardized coefficients; S. E., Standard Error; β, Standardized coefficients; *t* = *t*-test; VIF, Variance Inflation Factor; Adj. R^2^, Adjust R-squared; *P < 0.05.

**Table 6:** Summary table of hypotheses or findings.

Hypotheses	Direction of the relationship		Statistical values	Support of hypothesis
H1	General characteristics	Age ➔ Turnover	*β* = −0.22	Support (*P* = 0.048*)
H2	General characteristics	Educational level ➔ Turnover	*β* = 0.19	Support (*p* = 0.014*)
H3	Professional self-concept ➔ Turnover		*β* = −0.19	Support (*p* = 0.023*)
H4	Job stress ➔ Turnover		*β* = 0.53	Support (*p* < 0.001*)

β = Standardized coefficients; *P < 0.05.

## Discussion

The participants’ scores on professional self-concept, job stress, and turnover intention appeared mostly close to the median, similar to the studies of ward nurses by Lee and Jung (24) and Lee and Kim (25). Lee and Jung (24) focused on the professional self-concept, nursing work environment, and intimacy of nurses working in general hospitals. They found that as the nursing work environment improved and the professional self-concept of nurses and intimacy of nurses increased, the turnover intention decreased (7, 12, 26). In addition, Lee and Kim (25) mentioned that job stress, intimacy of nursing organizations, and emotional labor affected the turnover intention of ward nurses. This finding is associated with turnover intentions, suggesting that it is necessary to improve the nursing work environment to reduce the job stress of the nurses in dementia care centers by developing programs that enhance the relationship between nursing personnel and removing obstacles that undermine trust in the organization through continuous attention and efforts (27, 28). Previous research results (7, 12, 24–26) and Job Demands-Resources (JD-R) Theory or Self-Concept Theory are theoretically connected, and the results of this study are also consistent with the theoretical framework of this study, which is based on Job Demands-Resources (JD-R) Theory or Self-Concept Theory.

Next, the professional self-concept according to the general characteristics and job characteristics of the nurses in dementia care centers was higher as their total clinical experience increased. In addition, the job stress and turnover intention of the nurses in dementia care centers were higher in a younger age group (i.e., under the age of 25), whereas they were higher for nurses who had “graduate degrees or higher.” Overall, it is very likely that dementia care center nurses within the younger age range may have experienced role confusion as they simultaneously perform welfare services or administrative tasks in managing dementia patients, their families, and community members, which is beyond the scope of nurses’ original nursing duties (2, 4, 5). Having a heavy workload is a related factor as most nursing personnel working in dementia care centers perform their duties without distinction of roles. Also, the nurses in a younger age group seem to be unable to proficiently perform dementia care due to the lack of flexibility in responding to the situation and dealing with conflicts with dementia patients (4). However, previous studies (29, 30) contradict the results of this study as the subjects of the study were clinical nurses working in hospitals. Thus, further research is needed to support the present study’s findings on professional self-concept, job stress, and turnover intention according to the general characteristics and job characteristics of nurses in dementia care centers. Particularly, conducting repeated and expanded studies considering a different sample or subjects would be beneficial in understanding the critical factors affecting nurses’ turnover intention.

This study shows that the higher the tendency to have reduced professional self-concept and increased job stress, the higher the turnover intention of the nurses in dementia care centers. This research is similar with Kim and Park (31) who examined the relationship between the professional self-concept, clinical decision-making ability, and nursing work performance among nurse practitioners. Kim and Park (31) findings demonstrated that the more nurses in dementia care centers had autonomy in caring for dementia patients and performed independent nursing duties, the more accountability they had, the higher the value of professional nursing, and the better the job embeddedness was maintained. In that case, their job satisfaction was also higher, which naturally improved nursing work performance. Thus, these factors may be vital in the turnover intention of nurses working in dementia care centers. The results of this study, along with the results of this previous study (31), reinforce and are consistent with the theoretical foundation of this study, the Job Demands-Resources (JD-R) Theory or Self-Concept Theory.

Lastly, the effects on the turnover intention of the nurses working in dementia care centers were found in the following order: job stress, age, educational background, and professional self-concept. Accordingly, to adapt well to the nursing work at dementia care centers within the more complex community, the distribution between dementia care personnel and workload should be effective. The nurses in dementia care centers need to develop a positive professional self-concept, increase their autonomy, and actively provide nursing care (7, 32, 33). Nursing personnel in dementia care centers need to be rearranged according to the number of dementia patients by restructuring the system in the center. Welfare benefits, such as promotion opportunities, salary level increases, incentive, and risk allowances should be also provided through an appropriate compensation system according to the workability at the level of nursing organizations in dementia care centers (34, 35). These efforts can help improve the work performance of nurses in dementia care centers. More importantly, the nursing practice skills of nurses in dementia care centers would develop with such efforts and enhance job satisfaction. The results of this study and the results of these previous studies (32–35) support the theoretical basis of this study, Job Demands-Resources (JD-R) Theory or Self-Concept Theory.

Regarding the conceptual or theoretical advancement, based on the Job Demands-Resources (JD-R) Theory, even if job demands are high, employees can maintain their well-being if sufficient resources are provided. However, when job demands are high and job stress is high due to an imbalance between job demands and resources, the intention to change jobs increases and the employees change jobs. According to Self-Concept Theory, an individual’s self-concept can be formed and changed by the environment, experiences, and social interactions. Self-concept has a significant impact on an individual’s behavior, and a positive self-concept can lead to high achievement and well-being. On the contrary, a negative self-concept can lead to low achievement and dissatisfaction. Professional self-concept refers to how one perceives one’s self as a professional nurse working in the field. A negative professional self-concept leads to low achievement in nursing work and high dissatisfaction with the nursing profession, which increases turnover intention and leads to turnover. The results of this study further strengthen the Job Demands-Resources (JD-R) Theory or Self-Concept Theory and provide theoretical contributions by supporting these theories. The results of this study, which showed that job stress and professional self-concept significantly affect nurses’ ability to maintain professional nursing in a community dementia care setting, were consistent with and supported existing knowledge and theories.

### Implications for practice, policy, and research

The study’s findings imply that it is necessary to create a nursing work environment that can reduce the job stress of nurses in dementia care centers and lessen their turnover intention. It is also essential to provide systematic programs to cultivate the abilities and qualities of professionals for each dementia care center department and apply a competency-based personnel system fairly and transparently to improve nurses’ professional self-concept. The welfare and an appropriate compensation system of nurses in dementia care centers should constantly be prioritized as the scope of work of dementia care center nurses expands due to the growing aging population in Korea, leading to a rapid increase in the number of dementia patients. This study can be used as a reference for understanding aspects linked to reducing nurses’ turnover rate and promoting evidence-based personnel management of nurses working in dementia care centers. Further longitudinal studies considering other vital factors related to the turnover intention of nurses in dementia care centers by department, reflecting Korea’s characteristics, need to be conducted. An in-depth qualitative study that considers the causes and circumstances of the turnover rates of nurses in dementia care centers and the related environmental factors and personal characteristics may be carried out.

### Limitations

Studies related to nurses at dementia care centers in community are still insufficient, and the study participants were nurses at dementia care centers located in some metropolitan cities, South Korea. Therefore, due to limitations in sampling, there are limitations to explaining the impact on turnover intention of nurses at all dementia care centers in South Korea.

This study is relevant as it presents fundamental data on the work performance of nurses providing dementia care in Korea by investigating the relationship between their professional self-concept and job stress and their effect on turnover intention.

## Conclusion

This study found that the higher the professional self-concept, the lower the tendency to have job stress and turnover intention. Moreover, job stress, age, educational background, and professional self-concept (with an explanatory power of 21%) were observed as influencing factors on the turnover intention of nurses in dementia care centers. It was also discovered that the higher the job stress, the younger the age, the higher the educational level, and the lower the professional self-concept, the higher the turnover intention is to leave the job. The results of this study can serve as a basis for reducing the turnover intention of nurses working in dementia nursing centers and ultimately providing high-quality nursing care.

## Data Availability

The original contributions presented in the study are included in the article/Supplementary material, further inquiries can be directed to the corresponding author.
